# Effectiveness of vitamin D for irritable bowel syndrome: A protocol for a systematic review of randomized controlled trial

**DOI:** 10.1097/MD.0000000000014723

**Published:** 2019-03-01

**Authors:** Sheng-Mei Shi, Yan-Li Wen, Hai-Bin Hou, Hai-Xia Liu

**Affiliations:** a Department of Gastroenterology; b Department of Orthopedics, Yanan University Affiliated Hospital, Yan’an; c Department of Gastroenterology, Baoji Central Hospital, Baoji, China.

**Keywords:** effectiveness, irritable bowel syndrome, randomized controlled trial, safety, systematic review, vitamin D

## Abstract

**Background:**

Irritable bowel syndrome (IBS) is a prevalent and debilitating condition for patients who experience this disorder. Clinical researches indicate that vitamin D (VD) can help relief the symptoms of IBS. However, no systematic review has addressed this issue yet. Thus, this systematic review aims to investigate the effectiveness and safety of VD for patients with IBS.

**Methods:**

We will retrieve the following databases for randomized controlled trials to assess the effectiveness and safety of VD for patients with IBS: Cochrane Library, EMBASE, MEDICINE, Web of Science, Allied and Complementary Medicine Database, Chinese Biomedical Literature Database, and China National Knowledge Infrastructure. Each database will be retrieved from its inception to January 31, 2019. Two researchers will independently selection studies, extract data and assess methodological quality. RevMan 5.3 software will be used to pool the data, and carry out the meta-analysis if it is possible.

**Results:**

This systematic review will evaluate the effectiveness and safety of VD for patients with IBS. The primary outcomes include stool frequency and abdominal pain. The secondary outcomes consist of stool status, quality of life, and adverse effects.

**Conclusions:**

The findings of this systematic review may provide the existing evidence on the effectiveness and safety of VD for patients with IBS.

**Ethics and dissemination:**

This systematic review will not require ethical approval, because all data will be extracted from the published literature. The findings of this study will be disseminated at peer-reviewed journals.

**PROSPERO registration number:** PROSPERO CRD42019122641.

## 1 Introduction

Irritable bowel syndrome (IBS) is a very common chronic gastrointestinal condition.^[1,2]^ It often manifests as abdominal pain, bloating, and altered bowel habit, including constipation and diarrhea.^[3–5]^ Its prevalence was reported about approximately 4.6% to 21.2% in adults in Asia,^[6,7]^ and 10% to 15% in North America and Europe.^[8]^ Additionally, it is most common occurred between 20 and 40 years of age with a significant female predominance.^[9]^

Numerous managements are reported to treat this disorder,^[4,10–11]^ such as medication, dietary, yoga, acupuncture, moxibustion, electrical stimulation, and vitamin D (VD).^[12–19]^ Although lots of studies have reported that VD is effective for IBS,^[20–26]^ its effectiveness and safety is still not conclusive in patients with IBS. In addition, no systematic review regarding this topic has been conducted previously. Thus, this systematic review will evaluate the effectiveness and safety of VD for patients with IBS.

## 2 Methods and analysis

### 2.1 Study registration

This systematic review protocol has been registered on PROSPERO (CRD42019122641), and it has been reported following the Preferred Reporting Items for Systematic Reviews and Meta-Analysis Protocol statement guidelines.^[27]^

## 3 Eligibility criteria for study selection

### 3.1 Study types

This study will only consider randomized controlled trials (RCTs) of VD for IBS. All other studies will be excluded, such as Non-RCTs, quasi-RCTs, case studies, cross-over studies, and non-clinical studies.

### 3.2 Intervention types

The experimental intervention includes any forms of VD. However, the combination of VD with other therapies will not be considered. The control treatment can include any therapies except VD.

### 3.3 Patient types

Patients with IBS, regardless the race, sex, and age will be fully considered for inclusion in this study.

### 3.4 Outcome measurements

#### 3.4.1 Primary outcome

Stool frequency (as measured by average daily, or weekly or monthly stool frequency); abdominal pain (as assessed by any pain scales, such as visual analog scale).

#### 3.4.2 Secondary outcome

Stool status (as measured by Bristol scale); Quality of life (as evaluated by IBS Quality of Life Questionnaire, or any other instruments); adverse effects (any expected and unexpected adverse reactions or effects).

### 3.5 Search strategy

#### 3.5.1 Electronic databases search

We will search the following electronic bibliographic databases for relevant studies without any language restrictions: Cochrane Library, EMBASE, MEDICINE, Web of Science, Allied and Complementary Medicine Database, Chinese Biomedical Literature Database, and China National Knowledge Infrastructure. All databases will be searched from their inceptions to the January 31, 2019. The sample of detailed search strategy for Cochrane Library is shown in Table 1. Similar search strategies for other electronic databases will also be applied.

**Table 1:**
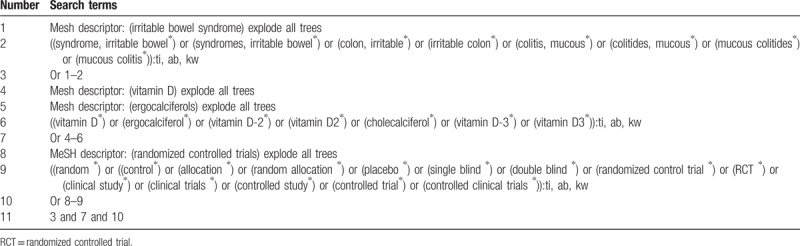
Search strategy applied in Cochrane library database.

#### 3.5.2 Other literature sources search

We will also search reference lists of relevant reviews and conference proceedings to avoid missing any potential eligible trials.

### 3.6 Study selection

**Figure 1:**
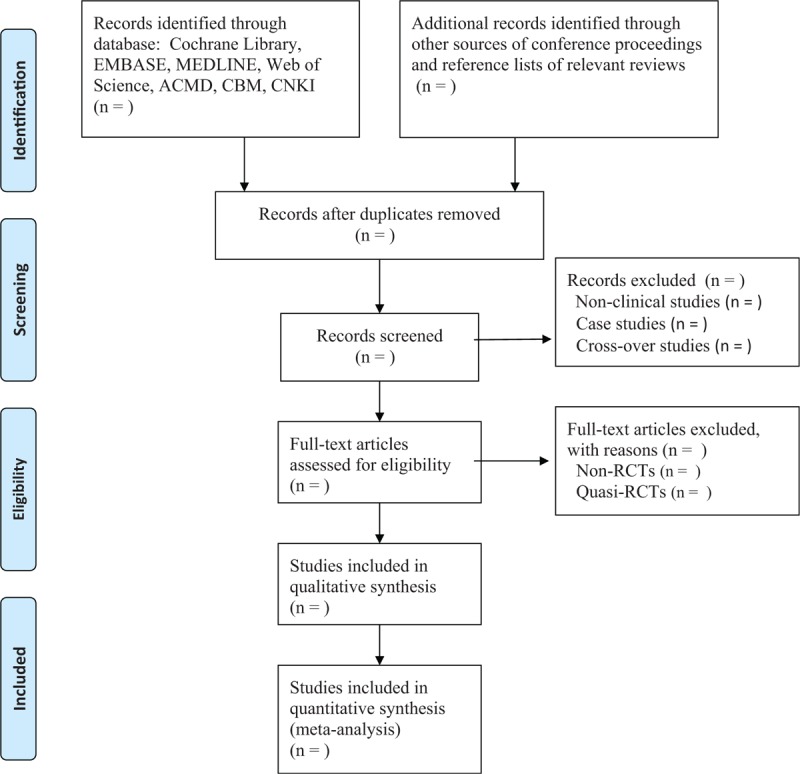
Process of study selection.

Two researchers will independently carry out the study selection by scanning titles and abstracts initially, and then reading the full-texts. They select all the studies according to the predefined eligibility criteria. The whole process of study selection is expressed in Figure 1. If there are disagreements about the study selection between 2 researchers, they will be solved by consulting a third experienced researcher.

### 3.7 Data collection and management

Two researchers independently extract the data based on the predefined data extraction sheet, which comprises of the following information.

Generation information: title, first author, published year, race, age, diagnostic criteria, inclusion, and exclusion criteria.

Study methods: sample size, randomization method, concealment, blinding, incomplete report, and any other sources of bias.

Intervention details: intervention names, dosage, frequency, and duration.

Outcomes: primary, secondary, and any other outcome measurements, such as adverse effects.

Endnote 7.0 software will be used to manage all the extracted data. If there are any divergences regarding the data extraction between 2 researchers, they will be solved by consulting a third experienced researcher.

### 3.8 Missing data deal with

Any insufficient, or missing data will be contacted the primary authors. If the authors fail to reply us, then only available data will be analyzed. In addition, the potential impacts of missing data will also be discussed.

### 3.9 Risk of bias assessment

Two researchers will independently assess the methodological quality for each included study by using Cochrane risk of bias tool on 7 aspects. Each aspect will be assessed as a high risk of bias, or unclear risk of bias, or low risk of bias. If there are any disagreements between 2 researchers, a third experienced researcher will be involved to solve them through discussion.

### 3.10 Treatment effect measurement

In this study, all continuous data will be reported as mean difference or standardized mean difference and 95% confidence intervals (CIs). All the dichotomous data will be expressed as risk ratio and 95% CIs.

## 4 Data analysis

We will utilize RevMan 5.3 software to analyze the data. The heterogeneity will be checked by using *I*^*2*^ test. We will apply a fixed-effect model to pool the data if fairly heterogeneity will be checked (*I*^*2*^ ≤50%). Otherwise, a random-effect model will be used to pool the data, if significant heterogeneity will be detected (*I*^*2*^ >50%). We will also carry out subgroup analysis to check any potential reasons that may result in heterogeneity. It will be conducted based on the different characteristics, treatments and outcome measurements. Meta-analysis will be performed if the heterogeneity is fairly well after the subgroup analysis. On the other hand, if the heterogeneity is still significant after the subgroup, we will not pool the data, and meta-analysis will not be performed under such situation.

Additionally, sensitivity analysis will also be conducted to check the robustness of pooled results by removing studies with high risk of bias. If sufficient eligible RCTs (normally more than 10 trials) are included, the funnel plot and Egg's regression test will be performed for checking the publication bias.^[28,29]^

## 5 Discussion

Previous clinical trials have hypothesized that VD plays a very important role in the treatment of IBS. However, no systematic review has addressed the effectiveness and safety of VD for IBS, and thus it is still at the conceptual level. Considering numerous literature on VD for IBS^[20–26]^, we will carry out a systematic review to inform the effectiveness and safety of VD in patients with IBS. The findings of this study are expected to provide up-to-date evidence regarding the effectiveness and safety of VD for IBS. In addition, the results of this study may also provide important evidence for either clinician and patients or health policy makers.

## Author contributions

**Conceptualization:** Sheng-Mei Shi, Hai-Bin Hou, Hai-Xia Liu.

**Data curation:** Sheng-Mei Shi, Yan-Li Wen, Hai-Xia Liu.

**Formal analysis:** Sheng-Mei Shi, Yan-Li Wen, Hai-Bin Hou.

**Funding acquisition:** Hai-Xia Liu.

**Investigation:** Hai-Bin Hou, Hai-Xia Liu.

**Methodology:** Sheng-Mei Shi, Yan-Li Wen, Hai-Bin Hou.

**Project administration:** Hai-Xia Liu.

**Resources:** Sheng-Mei Shi, Yan-Li Wen, Hai-Bin Hou.

**Software:** Sheng-Mei Shi, Yan-Li Wen, Hai-Bin Hou.

**Supervision:** Hai-Xia Liu.

**Validation:** Sheng-Mei Shi, Hai-Xia Liu.

**Visualization:** Yan-Li Wen, Hai-Bin Hou.

**Writing – original draft:** Sheng-Mei Shi, Yan-Li Wen, Hai-Xia Liu.

**Writing – review & editing:** Sheng-Mei Shi, Yan-Li Wen, Hai-Bin Hou, Hai-Xia Liu.
